# Genomic sequencing and analysis of a Chinese hamster ovary cell line using Illumina sequencing technology

**DOI:** 10.1186/1471-2164-12-67

**Published:** 2011-01-26

**Authors:** Stephanie Hammond, Jeffrey C Swanberg, Mihailo Kaplarevic, Kelvin H Lee

**Affiliations:** 1 Department of Chemical Engineering, University of Delaware, Newark, DE 19711, USA; 2 Delaware Biotechnology Institute, University of Delaware, Newark, DE 19711, USA

## Abstract

**Background:**

Chinese hamster ovary (CHO) cells are among the most widely used hosts for therapeutic protein production. Yet few genomic resources are available to aid in engineering high-producing cell lines.

**Results:**

High-throughput Illumina sequencing was used to generate a 1x genomic coverage of an engineered CHO cell line expressing secreted alkaline phosphatase (SEAP). Reference-guided alignment and assembly produced 3.57 million contigs and CHO-specific sequence information for ~ 18,000 mouse and ~ 19,000 rat orthologous genes. The majority of these genes are involved in metabolic processes, cellular signaling, and transport and represent attractive targets for cell line engineering.

**Conclusions:**

This demonstrates the applicability of next-generation sequencing technology and comparative genomic analysis in the development of CHO genomic resources.

## Background

With over half of all recombinant therapeutic proteins produced in mammalian cell lines, Chinese hamster ovary (CHO) cells remain the predominant production system for glycosylated biopharmaceuticals [1]. Although improvements in cell engineering, cell line selection, and culture conditions have increased productivity levels [2], the genetic basis underlying hyperproductivity remains poorly defined. The further development of genomic resources will facilitate detailed studies of genome structure, gene regulation, and gene expression in high-producing cell lines and aid in the use of sequence-specific molecular tools in cell line development.

A number of resources are required to support the assembly and annotation of the CHO genome including physical maps, genomic sequences, expressed sequence tag (EST) sequences, and proteomic data. Recent efforts to sequence and characterize bacterial artificial chromosome (BAC) libraries derived from CHO cells provide information for physical mapping of the CHO genome [3,4]. Transcriptomic and proteomic studies are currently used to examine differential expression of high-producing cell lines and to identify gene candidates for host cell engineering [5-7]. Transcriptomic studies which rely on cross-hybridization to mouse DNA microarrays showed some success [8,9], but also demonstrated the need for CHO-specific sequence information. Continued EST sequencing of CHO cells lines has generated databases containing more than 60,000 sequences and allowed for the development of CHO-specific DNA microarrays [10,11]. Furthermore, mapping of CHO EST sequences to a mouse genomic scaffold can potentially reveal structural and regulatory features of the CHO genome [12]. Such studies are limited in that only a subset of genes expressed at sufficiently high levels are captured for sequence analysis, providing little information regarding genome structure or non-transcribed portions of the genome.

At present, there is little genomic sequence data available for CHO cells. This limits the application of high-throughput molecular tools in gene discovery and cell line engineering. CHO cell lines also undergo multiple genomic rearrangements during the generation of high-producing cell lines, necessitating the sequencing of individual cell lines rather than the Chinese hamster [13,14]. Until recently, EST sequences were obtained by traditional Sanger technology [15], but current efforts are employing next-generation sequencing technologies including 454 and Illumina [11,16,17]. 454 pyrosequencing can generate up to 1 Gb of data in a single run, producing average read lengths of 330 bp with an average error rate of 4%, although a major limitation of this technology is the resolution of homopolymer regions [18,19]. Illumina sequencing can produce up to 90 Gb of data in a single run, generating reads up to 100 bp in length with an average error rate of 1-1.5% [19,20]. These technologies have significantly improved sequencing throughput and decreased cost, making mammalian genome sequencing feasible [20].

In this work, Illumina sequencing technology was used to generate an initial genomic sequence library of a Chinese hamster cell line with the goal of making these data available to the community. Comparative genomic analysis of this library was used to identify and functionally classify assembled sequences that were aligned to mouse and rat genes. An initial ~ 1x coverage of the CHO cell genome provided CHO-specific sequence information for a large number of protein coding genes, including those from functional classes typically underrepresented in EST libraries. This demonstrates that even low coverage genomic sequencing studies of CHO cell lines can increase the amount of sequence information available for this cell line.

## Results

### Illumina sequencing and reference-guided alignment

Gene-amplified CHO-SEAP cells expressing human secreted alkaline phosphatase (SEAP) were previously generated from CHO-DUK cells as a model for heterologous protein production [21]. Initial sequencing of the CHO-SEAP genome using Illumina technology yielded 2.72 Gb of genomic sequence, which represents a ~ 1x coverage of the CHO genome, estimated to be 2.8 Gb in size [4]. Reference-guided alignment was utilized in this study because a 1x genomic coverage is insufficient for *de novo *genome assembly. Previous work suggests that mouse and rat show a high degree of DNA sequence homology with the Chinese hamster [15] and several transcriptomic studies have employed a comparative approach to examine and annotate CHO sequence data [12,16]. Therefore, these species were chosen for comparative analysis. Reads were mapped to the reference genomes using MAQ software, which stands for Mapping and Assembling with Qualities [22]. Nearly 9% of the total reads were aligned to both reference genomes, although a slightly higher number of reads were aligned to the mouse genome (Table 1). Each aligned read was assigned a mapping quality that indicates whether the read has a unique alignment or can be aligned to multiple positions in the genome [22]. Based on MAQ mapping qualities, 50% of the short reads from the raw data set were aligned to repetitive regions of the reference genomes (Table 1). In general, results from alignment to both mouse and rat reference genomes suggest that CHO genomic sequences are generally more similar to mouse genomic sequences, as previously demonstrated [15].

**Table 1:** Alignment of CHO short reads to mouse and rat reference genomes

Sequence Type	Mouse		Rat	
Total aligned	6,582,209	(8.71%)	6,281,589	(8.31%)
Aligned to unique regions	3,202,228	(4.47%)	3,046,308	(4.28%)
Aligned to repeat regions	3,379,981	(4.24%)	3,235,281	(4.03%)
Aligned to protein-coding genes*	2,678,662	(3.52%)	2,359,457	(3.12%)

* Pseudogenes, RNA genes, and genes on Y chromosome were excluded from this analysis. Genes include exons, introns, and 5'- and 3'-untranslated regions

**Figure 1:**
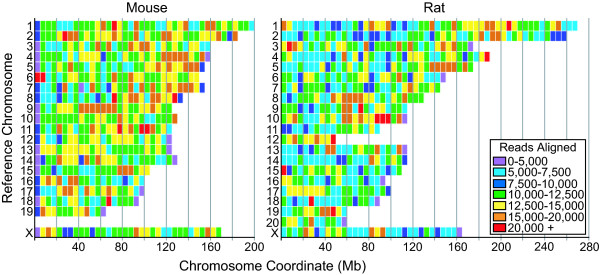
Distribution of CHO short reads along the mouse and rat reference genomes.

The distribution of CHO-SEAP reads aligned to the mouse and rat reference genomes was examined. The raw number of reads aligned was summed over 5 Mb bins along reference chromosomes (Figure 1). Reads were mapped along all reference chromosomes, although several regions of higher coverage are present in both reference genomes. Many chromosomes in the current build of the mouse genome contain gaps in the 5' end of the reference sequence, which may account for the low number of reads mapping to the chromosome ends.

**Figure 2:**
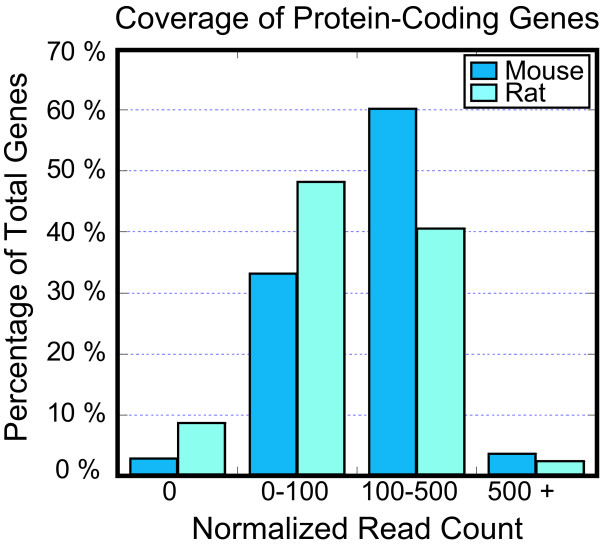
**Coverage of genes by short reads**. Normalized read counts of CHO sequences aligned to known protein-coding genes in the mouse and rat reference genomes. The raw number of short reads aligned to each gene is normalized by gene size. Gene coverage is classified as low, with fewer than 100 normalized read counts, medium, between 100-500 normalized read counts, or high, with greater than 500 normalized read counts.

Simulation studies based on Sanger technology suggest that most of the genomic coding sequence can be surveyed with less than 2-fold coverage [23] and low-coverage sequencing studies using Sanger [24] and next-generation [25,26] sequencing technologies are successful in producing partial sequences of orthologous genes. Nearly 40% of the aligned CHO reads are mapped to protein-coding mouse and rat genes (Table 1). Sequence information was collected for 97% of known protein-coding genes in the mouse genome and 93% of known protein-coding genes in the rat genome. To further examine gene coverage by this data set, the number of reads aligned to each gene in the mouse and rat protein-coding gene sets was examined (Figure 2). The raw read count was normalized by gene size to generate a normalized read count for each gene. This allows for a better comparison between the number of reads aligned to both very small, from thousands of bases, and very large, to millions of bases, genes. Only 3% of mouse genes and 7% of rat genes showed no coverage. Most genes showed low to medium coverage by the CHO short reads, with 33% of mouse and 48% of rat genes showing low coverage and 60% of mouse and 40% of rat genes showing medium coverage. A small proportion of both mouse and rat genes, less than 5%, showed a high level of coverage. Because CHO-SEAP cells are engineered to express high levels of a vector containing SEAP and dihydrofolate reductase (DHFR), the coverage of orthologous placental alkaline phosphatase and dihydrofolate reductase genes was examined. The placental-like alkaline phosphatase gene, *Alppl2*, showed medium to high coverage with 382 normalized read counts in rat and 582 in mouse. The *DHFR *gene showed low coverage, with normalized read counts of 67 in mouse and 55 in rat. Genes with the highest coverage had 5,000 - 30,000 normalized read counts.

### Consensus sequence assembly and analysis

To retrieve CHO sequences from this dataset, MAQ was used to assemble consensus genomic sequences of the mouse and rat alignments using sequence overlap information. In addition, performing the genomic assembly increased the length of the CHO sequences. Because of the 1-fold coverage of the 2.5 Gb mouse genome [27] and 2.75 Gb rat genome [28], a large proportion of the assembled sequences consisted of unsequenced bases, represented as N's in the consensus sequence. These files were parsed to extract CHO sequence contigs that range in size from 36 to 1,900 bp and have an average length of 54 bp, representing a 50% increase in length over the 36 bp short reads. A total of 1.86 million contigs from alignment to the mouse genome and 1.71 million contigs from alignment to the rat genome were produced. The total combined length of CHO contigs from alignment to the mouse genome was 101.6 million bases and from alignment to the rat genome was 91.4 million bases. This corresponds to a 32-36% coverage of the 2.8 Gb CHO genome. While 91% of these contigs are still short sequences less than 100 bp in length, over 9% exceed 100 bp in length and a small fraction are larger than 500 bp (Table 2). The GC content of these contigs was analyzed. Rodent genomes show a higher average genomic GC content, 42% for mouse and slightly higher for rat, compared to an average genomic GC content of 41% for human [27,28]. Overall, CHO contigs generated from alignment to the mouse and rat genomes show an average GC content of 43.0% and 42.9%, respectively. The GC content increases as contig length increases (Table 2).

**Table 2:** Summary of CHO contigs extracted from consensus sequences

			Contig Size					
			
Reference	Total		< 100 bp		100 to 500 bp		> 500 bp	
	Size (bp)	% GC	Size (bp)	% GC	Size (bp)	% GC	Size (bp)	% GC
Mouse	54	43.0	46	42.9	140	44.0	637	45.2
Rat	53	42.9	46	42.8	139	44.2	616	46.5

The average size and average GC content of CHO contigs generated from reference-guided alignment

This assembly set was annotated by comparing CHO contigs against custom genomic databases using a basic local alignment search tool (BLAST) algorithm [29]. Approximately 30% of these contigs resulted in significant matches in the genomic databases (Table 3). The high average similarity of these hits, ~ 96%, was expected due to the stringent alignment criteria, including the low number of allowed mismatches. Contigs that resulted in BLAST hits ranged in size from 36 to 1,000 bp, with an average size of 60 bp. A total of 563,163 contigs resulted in BLAST hits to non-protein coding regions of the genome, providing sequence information that is unavailable from EST sequencing experiments. An additional 479,270 contigs hit known protein-coding mouse and rat genes, providing CHO-specific sequence information for 17,883 mouse and 19,481 rat genes.

**Table 3:** Summary of assembly and BLAST analysis of CHO sequences

	Mouse	Rat
Total contigs	1,864,122	1,707,312
Contigs with BLAST hits	559,545	482,888
Average% similarity of BLAST hits	96.46%	96.39%
Contigs hit known genes	264,917	214,353
Total unique genes hit	17,883	19,481

**Figure 3:**
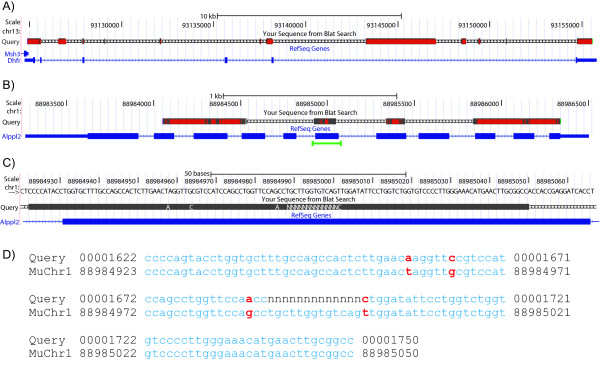
**Genomic landscape of CHO contigs**. CHO consensus sequences for A) *DHFR *and B) *Alppl2 *genes aligned to mouse reference chromosomes. Grey bars represent nucleotides identical to the reference sequence and red bars represent both nucleotides that differ from the reference sequence and gaps in the consensus sequences. CHO contigs aligned to an *Alppl2 *exon (highlighted in green in B) shown at the C) chromosome and D) nucleotide level. Red nucleotides indicate differences between the CHO consensus and reference sequences. Consensus sequences aligned to the mouse reference genome using BLAT [30] at the UCSC Genome Browser [31].

CHO contigs were examined in the context of the reference genomic structures. Regions within the consensus sequences were aligned to genes of interest in the mouse genome using BLAT [30] at the UCSC genome browser [31]. CHO contigs aligned to DHFR, the amplification marker, and placental alkaline phosphatase, the recombinant protein produced in this cell line, were chosen because of the interest in these genes for cell line development efforts. Contigs mapped within the coding regions of the mouse *DHFR *and *Alppl2 *genes are shown (Figure 3A-B). Two contigs of average size, 49 and 67 bp in length, separated by a small stretch of unsequenced bases mapped to an exon in *Alppl2 *(Figure 3C) and closer examination of this alignment reveals several nucleotide differences in the CHO contigs relative to the mouse reference sequence (Figure 3D).

**Figure 4:**
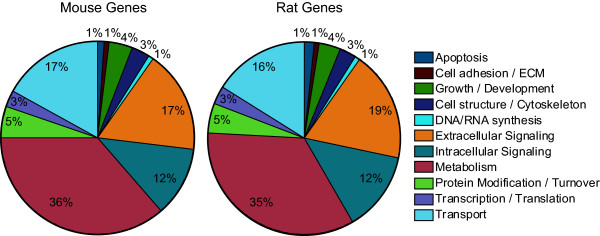
**Functional classification of genes identified in BLAST analysis**.

Gene ontology (GO) analysis was used to examine the functional classes of genes for which CHO sequences had been generated. GO terms were retrieved for the 17,883 mouse and 19,481 rat genes identified in the BLAST analysis of CHO contigs. A similar distribution of functional gene classes was observed for both mouse and rat assembly data sets (Figure 4). The top three functional groups identified in this analysis are genes associated with metabolism (~ 36%), intracellular and extracellular signaling (~30%) and transport (~ 17%). Although genes related to metabolic processes and vesicle-mediated transport are highly represented in EST sequencing libraries, genes involved in cellular signaling pathways are poorly represented [11]. Therefore, genomic sequencing of CHO cell lines provides additional sequence information on members of this and other functional classes that may be underrepresented in current EST libraries.

## Discussion

A comparative genomics approach was used to generate sequence-specific information for a high-producing CHO cell line with the goal of making this data publicly available. The development of CHO genomic resources will benefit not only cell line engineering efforts to enhance biopharmaceutical production but other areas of research utilizing CHO cells, such as the use of radiation hybrid mapping for comparative genomic analysis [32]. The analysis presented here demonstrates the potential of applying Illumina sequencing in the development of CHO genomic resources. Integration of genomic sequences derived from multiple next-generation technologies, such as 454 and Illumina sequencing, with those derived from Sanger sequencing enhance genomic coverage [33]. The inclusion of long paired-end or mate-paired libraries, with varied insert sizes, coupled with the high-throughput of next-generation sequencing technologies should also provide not only sequence but structural information required for *de novo *assembly of the CHO genome.

Neither the short reads nor the reference genomes were repeat-masked prior to alignment. A prevalent feature of mammalian genomes is the high content of repetitive sequences. Approximately 46% of the human genome, 37% of the mouse genome, and 40% of the rat genome are repetitive sequences [27,28]. Repeat-masking either the short reads or reference genome would discard information about a significant fraction of the genome and would reduce coverage in an uneven manner [22]. Recent work suggests that endogenous repetitive structures on CHO chromosomes may promote gene amplification and increase the stability of the amplified gene [3]. Including repetitive regions in the assembly and analysis may help identify genomic structures associated with hyperproductive CHO cell lines.

Several studies employed a similar approach to successfully generate genomic resources for non-model organisms from low-coverage data [25,34,35]. There are inherently some limitations to a reference-guided alignment and analysis regarding sequence similarity and genomic structure. MAQ allows up to 2 mismatches within the first 28 bp of each read and does not allow for gaps in the alignment [22]. Short reads derived from regions with less than 94% identity to the reference sequence may not be aligned [36]. This may account for the low percentage of total CHO reads aligned to either the mouse or rat genome and suggests that the CHO contigs presented here represent highly conserved regions between CHO cells and mouse or rat. In an initial genomic sequencing of the turkey using Illumina technology, only one-third of the short 35 bp reads could be directly aligned to the chicken genome, a closely related species, suggesting that a large portion of the short reads may not be expected to align in this type of analysis [34].

Additionally, during alignment, the sequenced genome is scaffolded onto the reference, so the structure of the final consensus sequence may not be representative of the true genomic architecture [36]. New methodologies are being developed to improve the consensus genomic sequences produced by reference-guided alignment [37,38]. CHO cell lines commonly used in biopharmaceutical production have a reduced chromosome number compared to primary Chinese hamster cells [4]. These cell lines also undergo genomic rearrangements as a result of amplification procedures used to develop high-producing cell lines [13,14]. Therefore, the genomic structure of the Chinese hamster may not be representative of the individual cell lines and analysis of specific CHO cell lines may provide a better understanding of the structural changes associated with hyperproductivity.

Of particular interest in CHO cell lines is examining the relationship between the location of the amplified gene and productivity of the cell line. BAC libraries were recently used to examine the site and structure of the transgene vector in gene-amplified cell lines [3,4]. The *DHFR *amplicon is large, up to several hundreds of thousands of nucleotides, and may contain repeated segments of the endogenous CHO genome [3,4,39]. The small lengths of the CHO contigs makes it unlikely that any contig will span both the *DHFR *amplicon and the host genome. Additionally, the transgene vector sequence is not present in the reference genome used during alignment. This makes it difficult to determine the integration site of the *DHFR *transgene vector in this analysis. A greater coverage of the CHO genome to permit *de novo *assembly of the reads will facilitate determining the integration site and copy number of the *DHFR *amplicon in this cell line. Increased coverage and refinement of the CHO genome will also enable detection of other copy number variants, such as insertions and deletions, and accurate SNP identification to assist cell line engineering efforts [40-42].

## Conclusions

The complexity of the CHO genome, including the structural rearrangements that occur during gene amplification and cell line derivation, makes assembly of a genomic sequence challenging. Next-generation sequencing technologies allow for the rapid acquisition of genomic sequence from CHO cell lines. This sequencing information can be used to generate a draft genome sequence when coupled with physical maps that can be derived from BAC libraries and a CHO scaffold that can be derived from cross-species comparative analysis. Incorporation of additional sequence data from transcriptomic studies and EST libraries will be necessary for complete annotation. The development of these resources is required to fully utilize sequence-specific tools, such as DNA microarrays and RNA interference, in cell line development and to understand how gene regulation and genome structure is altered in high-producing cell lines.

## Methods

### Genomic library construction and Illumina sequencing

CHO cells engineered to express human secreted alkaline phosphatase (SEAP) were generated from CHO-DUK cells (ATCC 9096) as described previously [21]. CHO-SEAP cells were maintained as adherent cultures in IMDM (Iscove's modified Dulbecco's medium, Invitrogen, Carlsbad, CA) supplemented with 10% dFBS (dialyzed fetal bovine serum, Invitrogen) and 5120 nM methotrexate (Calbiochem, San Diego, CA). Genomic DNA from CHO-SEAP cells was isolated using the Genomic DNA mini kit (Invitrogen). A single-end library was prepared using the DNA sample kit (Illumina, San Diego, CA) according to manufacturer's instructions. The genomic library was sequenced on an Illumina GA system at the Cornell University Life Sciences Core Laboratory Center (Ithaca, NY) by running 36 cycles according to manufacturer's instructions. Approximately 2.72 Gb from 75,583,814 high quality reads passed the Illumina GA Pipeline filter and were used for alignment. FASTQ files containing raw sequences and sequence qualities were deposited at the National Center for Biotechnology Information Sequence Read Archive (NCBI SRA) under the accession SRA012218. While analysis of SRA data sets is computationally challenging, rapid improvements in assembly algorithms and computational power are enabling more researchers to benefit from this type of data set.

### Sequence alignment and assembly

Reference genomes for mouse chromosomes 1-19 and X (M_musculus Build 37) and rat chromosomes 1-20 and X (R_norvegicus Build 3.4) were obtained from the NCBI genomic download site [43]. Reference guided alignment to both mouse and rat reference genomes and consensus sequence assembly was performed with MAQ 0.7.1 [22] using default settings. MapView was used for visual inspection of alignments [44]. Aligned reads were analyzed to determine if they mapped to unique or repetitive genomic regions, based on mapping qualities, or within protein-coding genes, based on genomic coordinates, using MATLAB (The MathWorks, Inc., Natick, MA). To verify sequencing reliability, the short read data set was aligned to the Chinese hamster mitochondrial genome (NC_007936.1) and resulted in significant homology.

### Gene coverage of protein-coding gene sets

Known protein-coding gene sets for both mouse and rat were established as follows: genomic coordinates for mouse and rat genes were retrieved from Mouse Genome Informatics (MGI) [45] and the Rat Genome Database (RGD) [46] and filtered to retain only known protein-coding genes. The mouse protein-coding gene sets contains 21,691 genes from chromosomes 1-19 and X and the rat protein-coding gene set contains 26,450 genes from chromosomes 1-20 and X. Gene size is defined from the genomic coordinates from MGI and RGD and includes exons, introns, and untranslated regions.

Normalized read counts were calculated for each gene in the protein-coding gene sets to which short reads were mapped. A normalization factor was calculated by dividing the size of each gene by the average gene size in the protein-coding gene sets, with an average gene size of 44,862 bp for mouse and 34,186 bp for rat. Normalized read counts were determined by dividing the raw number of reads aligned to each gene by the normalization factor calculated for that gene.

### Functional analysis of genomic assembly

Gene names and Gene Ontology (GO) terms were assigned to all contigs that shared sequence similarity with known protein-coding mouse and rat genes. Contigs were extracted from consensus sequences using Python and custom scripts. Contigs were aligned to reference genomes using BLAT [30] and viewed using the UCSC Genome Browser [31]. Sequence comparisons were done using standalone BLAST from NCBI [29]. Custom genomic databases were generated from mouse and rat reference chromosomes. Contigs were mapped to these genomic databases using BLASTN with a significance threshold of e < 1^-10^. BLAST outputs were parsed using Perl scripts to retrieve the best hit for each contig. Gene names and GO terms were retrieved for each contig that hit a known protein-coding gene. GO IDs for mouse (NCBIM37) and rat (RGSC3.4) genes were retrieved from ENSEMBL (release 56) using BioMart [47]. GO analysis was performed using the CateGOrizer web tool [48].

### Authors' contributions

SH performed data analysis and drafted the manuscript, JCS prepared samples and participated in data analysis, MK performed data analysis, KHL conceived the project. All authors have read and approved the final manuscript. The corresponding author will gladly provide detailed information on the cluster hardware setup, analysis software version and parameters, upon request.

## References

[B1] Wurm FM Production of recombinant protein therapeutics in cultivated mammalian cells Nat Biotechnol 2004 22 1393 1398 10.1038/nbt1026 15529164

[B2] Kuystermans D Krampe B Swiderek H Al-Rubeai M Using cell engineering and omic tools for the improvement of cell culture processes Cytotechnology 2007 53 3 22 10.1007/s10616-007-9055-6 19003186 PMC2267617

[B3] Park JY Takagi Y Yamatani M Honda K Asakawa S Shimizu N Omasa T Ohtake H Identification and analysis of specific chromosomal region adjacent to exogenous Dhfr-amplified region in Chinese hamster ovary cell genome J Biosci Bioeng 2010 109 504 511 10.1016/j.jbiosc.2009.10.019 20347775

[B4] Omasa T Cao Y Park JY Takagi Y Kimura S Yano H Honda K Asakawa S Shimizu N Ohtake H Bacterial artificial chromosome library for genome-wide analysis of Chinese hamster ovary cells Biotechnol Bioeng 2009 104 986 994 10.1002/bit.22463 19575438

[B5] Pascoe DE Arnott D Papoutsakis ET Miller WM Andersen DC Proteome analysis of antibody-producing CHO cell lines with different metabolic profiles Biotechnol Bioeng 2007 98 391 410 10.1002/bit.21460 17461427

[B6] Nissom PM Sanny A Kok YJ Hiang YT Chuah SH Shing TK Lee YY Wong KT Hu WS Sim MY Philp R Transcriptome and proteome profiling to understanding the biology of high productivity CHO cells Mol Biotechnol 2006 34 125 140 10.1385/MB:34:2:125 17172658

[B7] Yee JC Gerdtzen ZP Hu WS Comparative transcriptome analysis to unveil genes affecting recombinant protein productivity in mammalian cells Biotechnol Bioeng 2009 102 246 263 10.1002/bit.22039 18726962

[B8] Ernst W Trummer E Mead J Bessant C Strelec H Katinger H Hesse F Evaluation of a genomics platform for cross-species transcriptome analysis of recombinant CHO cells Biotechnol J 2006 1 639 650 10.1002/biot.200600010 16892312

[B9] Yee JC Wlaschin KF Chuah SH Nissom PM Hu WS Quality assessment of cross-species hybridization of CHO transcriptome on a mouse DNA oligo microarray Biotechnol Bioeng 2008 101 1359 1365 10.1002/bit.21984 18814282

[B10] Bahr SM Borgschulte T Kayser KJ Lin N Using microarray technology to select housekeeping genes in Chinese hamster ovary cells Biotechnol Bioeng 2009 104 1041 1046 10.1002/bit.22452 19557832

[B11] Kantardjieff A Nissom PM Chuah SH Yusufi F Jacob NM Mulukutla BC Yap M Hu WS Developing genomic platforms for Chinese hamster ovary cells Biotechnol Adv 2009 27 1028 1035 10.1016/j.biotechadv.2009.05.023 19470403

[B12] Wlaschin KF Hu WS A scaffold for the Chinese hamster genome Biotechnol Bioeng 2007 98 429 439 10.1002/bit.21430 17390381

[B13] Derouazi M Martinet D Besuchet Schmutz N Flaction R Wicht M Bertschinger M Hacker DL Beckmann JS Wurm FM Genetic characterization of CHO production host DG44 and derivative recombinant cell lines Biochem Biophys Res Commun 2006 340 1069 1077 10.1016/j.bbrc.2005.12.111 16403443

[B14] Ruiz JC Wahl GM Chromosomal destabilization during gene amplification Mol Cell Biol 1990 10 3056 3066 2188107 10.1128/mcb.10.6.3056-3066.1990 PMC360670

[B15] Wlaschin KF Nissom PM Gatti Mde L Ong PF Arleen S Tan KS Rink A Cham B Wong K Yap M Hu WS EST sequencing for gene discovery in Chinese hamster ovary cells Biotechnol Bioeng 2005 91 592 606 10.1002/bit.20511 16003777

[B16] Birzele F Schaub J Rust W Clemens C Baum P Kaufmann H Weith A Schulz TW Hildebrandt T Into the unknown: expression profiling without genome sequence information in CHO by next generation sequencing Nucleic Acids Res 2010 38 3999 4010 10.1093/nar/gkq116 20194116 PMC2896516

[B17] Jacob NM Kantardjieff A Yusufi FN Retzel EF Mulukutla BC Chuah SH Yap M Hu WS Reaching the depth of the Chinese hamster ovary cell transcriptome Biotechnol Bioeng 2010 105 1002 1009 19882695 10.1002/bit.22588

[B18] Margulies M Egholm M Altman WE Attiya S Bader JS Bemben LA Berka J Braverman MS Chen YJ Chen Z Genome sequencing in microfabricated high-density picolitre reactors Nature 2005 437 376 380 16056220 10.1038/nature03959 PMC1464427

[B19] Shendure J Ji H Next-generation DNA sequencing Nat Biotechnol 2008 26 1135 1145 10.1038/nbt1486 18846087

[B20] Metzker ML Sequencing technologies - the next generation Nat Rev Genet 2010 11 31 46 10.1038/nrg2626 19997069

[B21] Hayduk EJ Lee KH Cytochalasin D can improve heterologous protein productivity in adherent Chinese hamster ovary cells Biotechnol Bioeng 2005 90 354 364 10.1002/bit.20438 15772946

[B22] Li H Ruan J Durbin R Mapping short DNA sequencing reads and calling variants using mapping quality scores Genome Res 2008 18 1851 1858 10.1101/gr.078212.108 18714091 PMC2577856

[B23] Bouck J Miller W Gorrell JH Muzny D Gibbs RA Analysis of the quality and utility of random shotgun sequencing at low redundancies Genome Res 1998 8 1074 1084 9799794 10.1101/gr.8.10.1074 PMC310787

[B24] Kirkness EF Bafna V Halpern AL Levy S Remington K Rusch DB Delcher AL Pop M Wang W Fraser CM Venter JC The dog genome: survey sequencing and comparative analysis Science 2003 301 1898 1903 10.1126/science.1086432 14512627

[B25] Rasmussen DA Noor MA What can you do with 0.1x genome coverage? A case study based on a genome survey of the scuttle fly Megaselia scalaris (Phoridae) BMC Genomics 2009 10 382 10.1186/1471-2164-10-382 19689807 PMC2735751

[B26] Bai X Zhang W Orantes L Jun TH Mittapalli O Mian MA Michel AP Combining next-generation sequencing strategies for rapid molecular resource development from an invasive aphid species, Aphis glycines PLoS One 2010 5 e11370 10.1371/journal.pone.0011370 20614011 PMC2894077

[B27] Waterston RH Lindblad-Toh K Birney E Rogers J Abril JF Agarwal P Agarwala R Ainscough R Alexandersson M An P Initial sequencing and comparative analysis of the mouse genome Nature 2002 420 520 562 10.1038/nature01262 12466850

[B28] Gibbs RA Weinstock GM Metzker ML Muzny DM Sodergren EJ Scherer S Scott G Steffen D Worley KC Burch PE Genome sequence of the Brown Norway rat yields insights into mammalian evolution Nature 2004 428 493 521 10.1038/nature02426 15057822

[B29] Camacho C Coulouris G Avagyan V Ma N Papadopoulos J Bealer K Madden TL BLAST+: architecture and applications BMC Bioinformatics 2009 10 421 10.1186/1471-2105-10-421 20003500 PMC2803857

[B30] Kent WJ BLAT--the BLAST-like alignment tool Genome Res 2002 12 656 664 11932250 10.1101/gr.229202 PMC187518

[B31] The UCSC Genome Browser http://genome.ucsc.edu

[B32] Murphy WJ Stanyon R O'Brien SJ Evolution of mammalian genome organization inferred from comparative gene mapping Genome Biol 2001 2 REVIEWS0005 10.1186/gb-2001-2-6-reviews0005 11423011 PMC138942

[B33] Quinn NL Levenkova N Chow W Bouffard P Boroevich KA Knight JR Jarvie TP Lubieniecki KP Desany BA Koop BF Assessing the feasibility of GS FLX Pyrosequencing for sequencing the Atlantic salmon genome BMC Genomics 2008 9 404 10.1186/1471-2164-9-404 18755037 PMC2532694

[B34] Kerstens HH Crooijmans RP Veenendaal A Dibbits BW Chin AWTF den Dunnen JT Groenen MA Large scale single nucleotide polymorphism discovery in unsequenced genomes using second generation high throughput sequencing technology: applied to turkey BMC Genomics 2009 10 479 10.1186/1471-2164-10-479 19835600 PMC2772860

[B35] Wernersson R Schierup MH Jorgensen FG Gorodkin J Panitz F Staerfeldt HH Christensen OF Mailund T Hornshoj H Klein A Pigs in sequence space: a 0.66X coverage pig genome survey based on shotgun sequencing BMC Genomics 2005 6 70 10.1186/1471-2164-6-70 15885146 PMC1142312

[B36] Dutilh BE Huynen MA Strous M Increasing the coverage of a metapopulation consensus genome by iterative read mapping and assembly Bioinformatics 2009 25 2878 2881 10.1093/bioinformatics/btp377 19542148 PMC2781756

[B37] Schneeberger K Hagmann J Ossowski S Warthmann N Gesing S Kohlbacher O Weigel D Simultaneous alignment of short reads against multiple genomes Genome Biol 2009 10 R98 10.1186/gb-2009-10-9-r98 19761611 PMC2768987

[B38] Gnerre S Lander ES Lindblad-Toh K Jaffe DB Assisted assembly: how to improve a de novo genome assembly by using related species Genome Biol 2009 10 R88 10.1186/gb-2009-10-8-r88 19712469 PMC2745769

[B39] Wurm FM Petropoulos CJ Plasmid integration, amplification and cytogenetics in CHO cells: questions and comments Biologicals 1994 22 95 102 10.1006/biol.1994.1015 7917238

[B40] Yoon S Xuan Z Makarov V Ye K Sebat J Sensitive and accurate detection of copy number variants using read depth of coverage Genome Res 2009 19 1586 1592 10.1101/gr.092981.109 19657104 PMC2752127

[B41] Medvedev P Stanciu M Brudno M Computational methods for discovering structural variation with next-generation sequencing Nat Methods 2009 6 S13 20 10.1038/nmeth.1374 19844226

[B42] Liao PY Lee KH From SNPs to functional polymorphism: The insight into biotechnology applications Biochem Eng J 2010 49 149 158 10.1016/j.bej.2009.12.021

[B43] NCBI Genomes FTP site ftp://ftp.ncbi.nih.gov/genomes/

[B44] Bao H Guo H Wang J Zhou R Lu X Shi S MapView: visualization of short reads alignment on a desktop computer Bioinformatics 2009 25 1554 1555 10.1093/bioinformatics/btp255 19369497

[B45] Mouse Genome Informatics http://www.informatics.jax.org

[B46] Rat Genome Database http://rgd.mcw.edu/

[B47] BioMart http://www.biomart.org

[B48] Zhi-Liang H Bao J Reecy JM CateGOrizer: A Web-Based Program to Batch Analyze Gene Ontology Classification Categories Online J Bioinformatics 2008 9 108 112

